# Beyond One-Size-Fits-All: Tailoring Teicoplanin Regimens for Normal Renal Function Patients Using Population Pharmacokinetics and Monte Carlo Simulation

**DOI:** 10.3390/pharmaceutics16040499

**Published:** 2024-04-05

**Authors:** Yong-Kyun Kim, Kyeong-Min Jo, Jae-Ha Lee, Ji-Hoon Jang, Eun-Jun Choe, Gaeun Kang, Dae-Young Zang, Dong-Hwan Lee

**Affiliations:** 1 Division of Infectious Diseases, Department of Internal Medicine, Hallym University Sacred Heart Hospital, Hallym University College of Medicine, Anyang 14066, Republic of Korea; amoureuxyk@hallym.or.kr; 2 Department of Infectious Diseases, Inje University Haeundae Paik Hospital, Inje University College of Medicine, Busan 48108, Republic of Korea; minfection@paik.ac.kr; 3 Department of Pulmonology and Critical Care Medicine, Inje University Haeundae Paik Hospital, Inje University College of Medicine, Busan 48108, Republic of Korea; anilleus@paik.ac.kr (J.-H.L.); h00596@paik.ac.kr (J.-H.J.); h00685@paik.ac.kr (E.-J.C.); 4 Division of Clinical Pharmacology, Chonnam National University Hospital, Gwangju 61469, Republic of Korea; bp00092@cnuh.com; 5 Division of Hematology-Oncology, Department of Internal Medicine, Hallym University Sacred Heart Hospital, Hallym University College of Medicine, Anyang 14066, Republic of Korea; fhdzang@hallym.or.kr; 6 Department of Clinical Pharmacology, Hallym University Sacred Heart Hospital, Hallym University College of Medicine, Anyang 14066, Republic of Korea

**Keywords:** teicoplanin, population pharmacokinetics, noncompartmental analysis, Monte Carlo simulation, norma renal function, healthy, adult

## Abstract

In patients with normal renal function, significant teicoplanin dose adjustments are often necessary. This study aimed to develop a population pharmacokinetic (PK) model for teicoplanin in healthy adults and use it to recommend optimal dosage regimens for patients with normal renal function. PK samples were obtained from 12 subjects and analyzed using a population approach. The derived parameters informed Monte Carlo simulations for dosing recommendations. The PK profile was best described using a three-compartment model, in which the estimated glomerular filtration rate calculated via the CKD-EPI equation and adjusted for body surface area was identified as a significant covariate affecting total clearance. For pathogens with a minimum inhibitory concentration of 1 mg/L, a loading dose (LD) of 14 mg/kg administered every 12 h for four doses, followed by a maintenance dose (MD) of 16 mg/kg administered every 24 h, is recommended. These findings indicate the need for dosage adjustments, such as increasing the LD and MD or decreasing the dosing interval of MD in patients with normal renal function. Because of the long half-life of teicoplanin and the requirement for long-term administration, therapeutic drug monitoring at strategic intervals is important to avoid nephrotoxicity associated with elevated trough concentrations.

## 1. Introduction

Teicoplanin, along with the glycopeptide antibiotic vancomycin, has an important role in the treatment of Gram-positive bacterial infections, including those caused by methicillin-resistant Staphylococcus aureus (MRSA) [1,2]. Because of its long elimination half-life ranging from 83 to 182 h in patients with normal renal function, teicoplanin requires a loading dose to achieve therapeutic concentrations [2]. Based on the European Medicines Agency’s summary of the product characteristics (SmPC) for teicoplanin, adults and the elderly with normal renal function have recommended specific doses to effectively treat Gram-positive infections [3]. For conditions such as complicated skin and soft tissue infections, pneumonia, and complicated urinary tract infections, a loading dose (LD) of 400 mg or 6 mg/kg every 12 h for three initial doses is recommended, followed by a daily maintenance dose (MD) of 6 mg/kg to achieve trough concentrations (C_trough_) greater than 10 mg/L, as determined via high-performance liquid chromatography (HPLC). For more severe conditions, such as endocarditis, a C_trough_ of 15–30 mg/L, an increased LD of 800 mg or 12 mg/kg every 12 h for the first three doses, followed by an MD of 12 mg/kg daily, is recommended. Despite the emphasis on achieving specific C_trough_ targets for therapeutic success, the pharmacokinetic/pharmacodynamic (PK/PD) relationship of teicoplanin suggests that the ratio of the area under the concentration–time curve (AUC) for 24 h to the minimum inhibitory concentration (MIC) may be a more appropriate marker to predict teicoplanin efficacy. As an antibiotic that exhibits time-dependent killing activity, this PK/PD index is important to correlate with drug efficacy [4]. Studies of patients infected with MRSA have concluded that an AUC/MIC of ≥610–900 is required for a sufficient bacteriological response [5,6,7,8].

Because of the complexity of calculating the AUC through multiple blood samplings, using C_trough_ as a surrogate marker for AUC is considered customary in clinical practice. However, the recently revised consensus guidelines for vancomycin, based on the opinions of experts in the field and several studies, have concluded that C_trough_ is not an adequate surrogate indicator for AUC [9]. The guidelines now recommend the use of Bayesian-derived AUC monitoring, using well-developed vancomycin population PK models integrated into Bayesian software. For teicoplanin, AUC may be a more accurate predictor of efficacy than C_trough_, but a consensus on this matter has yet to be established. Therefore, developing a robust PK model for teicoplanin and creating Bayesian software would greatly assist in the precision dosing of teicoplanin.

When developing new drugs, the evaluation of tolerance and PK in healthy individuals, who are not affected by the confounding factors of the disease, is undertaken to explore the basic characteristics of a drug. Similarly, calculating the standard PK parameters of a drug in population PK studies not only helps to understand the population PK in patients through subsequent patient-focused research, but also helps to create models with superior predictive power. The purpose of this study was to develop a population PK model for healthy adults and use it to predict the optimal dosage regimen in patients with normal renal function.

## 2. Materials and Methods

### 2.1. Subjects

The inclusion criteria for this study were as follows: (1) Individuals between 19 and 55 years old on the screening date; (2) without any congenital or chronic diseases, and no pathological symptoms or findings upon internal medical examination; (3) considered eligible based on health screening assessments, including medical history, vital signs, physical examination, hematological tests, blood chemistry, urinalysis, and serological tests for infections. The key exclusion criteria were as follows: (1) Individuals with clinically significant diseases or past medical history in the gastrointestinal, cardiovascular, respiratory, endocrine, hepatobiliary, hematologic, oncologic, musculoskeletal, renal, neurological, psychiatric, immunological, urological, ophthalmological, and otolaryngological systems, or those with genetic disorders; (2) a history that could affect the absorption, distribution, metabolism, or excretion of drugs, such as past liver or kidney diseases; (3) hypersensitivity or a history of hypersensitivity to teicoplanin; (4) positive serological test results for HBsAg, anti-HCV Ab, HIV Ag/Ab, or syphilis; (5) pregnant or breastfeeding individuals, or those with the possibility of being pregnant.

### 2.2. Study Design

A 200 mg dose of teicoplanin in 100 mL of normal saline was administered intravenously to subjects over 30 min. Venous blood samples (6 mL each) were collected into heparinized tubes at 33, 36, 45, and 90 min, and 4, 8, 48–120, and 168–240 h after starting the infusion. Eight sampling points were used per subject to analyze PK parameters via noncompartmental and population PK methods.

### 2.3. Drug Assay

Plasma levels of teicoplanin were quantified using tandem mass spectrometry coupled with HPLC (HPLC-MS/MS). The analytical system utilized included an LC-40 HPLC unit from Shimadzu Co. (Kyoto, Japan) paired with a Gemini C18 analytical column from Phenomenex (Torrance, CA, USA). A SCIEX 4500 QTRAP mass spectrometer (Sciex, Redwood City, CA, USA) facilitated the MS-based detection. Calibration standards were prepared by combining 100 μL of a reference solution with 10 μL of a vancomycin internal standard (concentration of 100 μg/mL) in microcentrifuge tubes. To this mixture, 400 μL of acetonitrile was added to precipitate proteins, followed by vortex mixing for 60 s. Following centrifugation at 12,000 rpm for 2 min at 4 °C, the clear supernatant was diluted 10-fold with deionized water. A 10 μL sample of this solution was then introduced into the HPLC-MS/MS for analysis. In a parallel procedure for plasma samples, after the addition of the vancomycin internal standard and acetonitrile-induced protein precipitation, the sample was processed like that of the calibration standard preparation before HPLC-MS/MS analysis. The teicoplanin concentration in the plasma was determined by comparing the peak area ratio of teicoplanin to the internal standard and employed a batch-specific calibration curve equation, adjusted for 1/x^2^ weighting.

### 2.4. Population Pharmacokinetic Analysis

The PK analysis of teicoplanin was completed using a nonlinear mixed-effects modeling technique with NONMEM software (version 7.5, ICON Clinical Research LLC, North Wales, PA, USA). Parameter estimation for both the observed and the unaccounted-for random effects was achieved by applying the First-Order Conditional Estimation with Interaction approach. This method facilitates the consideration of interactions between the unexplained interindividual variability (IIV) of PK parameters and the residual unexplained variability in the observed concentrations. To model the PK profiles of teicoplanin, we used one-compartment (ADVAN1 TRANS2), two-compartment (ADVAN3 TRANS4), and three-compartment (ADVAN11 TRANS4) models which were based on first-order kinetics, with the exception of zero-order infusion processes.

Model selection and evaluation were based on several criteria: NONMEM’s objective function values (OFVs), parameter estimate precision (reflected by relative standard errors), IIV shrinkage, diagnostic fit plots, visual predictive checks, and bootstrap analysis. For nested model comparisons, a decrease in OFV (ΔOFV) exceeding 3.84 (for a χ^2^ distribution with one degree of freedom, df) or 5.99 (for a χ^2^ distribution with two df) indicated meaningful improvements at a significance level of *p* < 0.05. The diagnostic plots for model assessment include conditional weighted residuals (CWRES) against time and population predictions (PRED), as well as actual observations compared with both PRED and individual predictions. Visual predictive checks (VPC) were conducted by matching the observed concentrations to the 80% prediction intervals from 1000 simulations based on the final PK model. The variability in the final predictions was determined by calculating the median and 95% confidence intervals for the PK parameters from 2000 bootstrap samples.

Significant covariates affecting PK parameters were determined using stepwise selection, with inclusion at *p* < 0.01 (ΔOFV < −6.635) and exclusion at *p* < 0.001 (ΔOFV > 10.83) for one df. Covariates had to show statistical and clinical relevance. We analyzed demographic (gender, age, weight, height, body mass index, body surface area) and laboratory (serum proteins, albumin, creatinine, cystatin C levels) factors. In addition, the effect of renal clearance on teicoplanin clearance, estimated through the Cockcroft–Gault, MDRD, and CKD-EPI formulas, was examined.

For covariate identification, model evaluation through VPCs, and the execution of nonparametric bootstrapping, the Perl-speaks-NONMEM (PSN, version 5.3.1) tool was used. The R programming environment (version 4.3.2) was also used for the post-analysis processing and graphical representation of the outcomes.

### 2.5. Noncompartmental Analysis

A noncompartmental analysis (NCA) was completed to evaluate the plasma concentration–time profiles of teicoplanin using the R programming language [10] and the NonCompart package [11]. The following PK parameters were assessed: maximum observed plasma concentration (C_max_), time of last measurable concentration (T_last_), concentration corresponding to T_last_ (C_last_), area under the plasma concentration–time curve (AUC) from the start of dosing to the last quantifiable concentration (AUC_last_), AUC from the start of dosing to infinity (AUC_inf_), area under the first moment curve (AUMC) from 0 h to the T_last_ (AUMC_last_), AUMC extrapolated to infinity, based on the last observed concentration (AUMC_inf_), mean residence time from 0 h to infinite (MRT_inf_), total body clearance as determined through NCA (CL_NCA_), volume of distribution (Vd) determined via NCA (V_zNCA_), steady-state Vd determined via NCA (V_ssNCA_), and terminal elimination half-life (t_1/2λz_). C_max_, T_last_, and C_last_ were determined directly from the observed data. AUC_last_ and AUMC_last_ were calculated employing the linear-up and log-down trapezoidal method. AUC_inf_ was estimated by adding C_last_/λ_z_ to AUC_last_, where λ_z_ is the terminal elimination rate constant, which was determined by log-linear regression of the terminal phase plasma concentrations. AUMC_inf_ was calculated using the following formula: AUMC_last_ + (T_last_ × Clast)/λ_z_ + Clast/λ_z_^2^, MRT_inf_ as AUMC_inf_/AUC_inf_—infusion time/2, CL_NCA_ as dose/AUC_inf_, V_zNCA_ as CL_NCA_/λ_z_, V_ssNCA_ as MRT_inf_ × CL_NCA_, and t_1/2λz_ as ln(2)/λ_z_.

### 2.6. Dosage Simulations

To develop dosage recommendations for teicoplanin in patients with normal renal function, we used Monte Carlo simulations based on the final PK model. Building upon this model, we generated PK parameters for 5000 virtual patients to assess the therapeutic targets for teicoplanin. The analysis focused on two primary endpoints obtained from days 3 to 7 following the initiation of treatment: the C_trough_, which was determined just before the next dose, and the ratio of the AUC from 0 to 24 h to the pathogen’s MIC, which was designated AUC/MIC. The simulation process involved two distinct strategies. Initially, PK profiles were simulated following the administration of LDs of 6, 8, 10, 12, 14, and 16 mg at 12 h intervals for four doses, followed by MDs at 24 h intervals for five doses. In another set of simulations, the MDs were administered every 12 h for 10 doses, adhering to the same initial loading regimen.

To evaluate the potential therapeutic efficacy of teicoplanin, we conducted two analyses. The first analysis calculated the probability of target attainment (PTA), in which C_trough_ exceeded specified targets of 10, 15, 20, and 30 mg/L. In the second analysis, MIC values of 0.25, 0.5, 1, and 2 mg/L were randomly assigned to 5000 virtual patients, reflecting the distribution of teicoplanin MICs for MRSA as reported by the European Committee on Antimicrobial Susceptibility Testing (EUCAST) [12]. The distribution of MIC values among the virtual patients was as follows: 0.25 mg/L for 5.6% (31/555), 0.5 mg/L for 40.4% (224/555), 1 mg/L for 43.1% (239/555), and 2 mg/L for 11.0% (61/555) of the cases. Based on these distributions, we calculated the PTA, in which the C_trough_ was at least 20 mg/L and, concurrently, the AUC/MIC ratio was at least 800. For all simulation scenarios performed in this study, encompassing various combinations, the proportion of instances, in which C_trough_ exceeded 60 mg/L, considered as a marker of nephrotoxicity [13,14], was calculated individually for each case.

## 3. Results

### 3.1. Subjects

The demographic and clinical characteristics of the 12 healthy adult subjects (6 females, 6 males) are listed in Table 1. The median (interquartile range) estimated glomerular filtration rate (eGFR) calculated using the CKD-EPI formula with creatinine levels was 105 (97.9–115) mL/min/1.73 m^2^, and 108 (98.8–120) mL/min/1.73 m^2^ using both creatinine and cystatin C levels with the CKD-EPI formula. The eGFRs calculated using MDRD and the two CKD-EPI formulas were adjusted for the body surface area (BSA) of each subject and are listed in Table 1.

### 3.2. Population Pharmacokinetic Analysis

A total of 96 plasma samples were used for this analysis. The time course of teicoplanin concentrations was best described through a three-compartment PK model (Table 2). The OFVs for the one-, two-, and three-compartment basic models were 305.997, −65.658, and −186.520, respectively. The three-compartment model was characterized using parameters including total clearance (CL), volume of distribution in the central compartment (V1), distribution volumes for the first (V2) and second (V3) peripheral compartments, along with intercompartmental clearances (Q2 between V1 and V2, as well as Q3 between V1 and V3). For the final PK model, which had an OFV of −209.055, the GFR, as estimated via the CKD-EPI equation using creatinine levels, was a significant factor that affected total clearance (CL). Removing this eGFR covariate from the model resulted in an increased OFV to −189.463, thus indicating its importance. In addition, body weight significantly influenced the V3, with the OFV rising to −192.447 in a model excluding the effect of weight on V3. The BSV for CL, V1, V2, and V3 were fixed due to their relative standard error (RSE) exceeding 25%.

**Figure 1:**
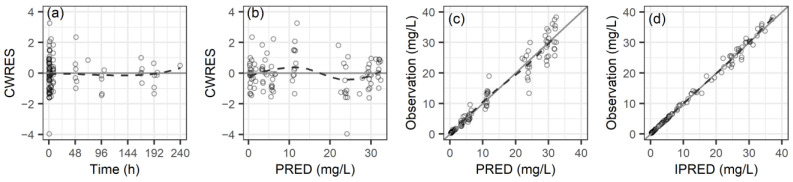
Diagnostic plots for the final PK model of teicoplanin: (**a**) conditional weighted residuals (CWRES) versus time, (**b**) CWRES versus population predicted concentration (PRED) (**c**) observed concentration versus PRED, and (**d**) observed concentration versus individual predicted concentration (IPRED). The dashed lines are smooth curves.

The goodness-of-fit diagnostics for the concluding PK model are illustrated in Figure 1. The distribution of CWRES and observed concentrations closely aligns with the x-axis or unity line, suggesting minimal bias in the PK parameters and affirming the model’s adequacy. The individual fit plots for teicoplanin are shown in Figure S1. The VPC for the teicoplanin PK model is presented in Figure S2, in which the observed data’s 10th, 50th, and 90th percentiles largely fall within the 95% confidence interval for the simulated data. This demonstrates the model’s robust predictive accuracy and its effective encapsulation of the observed concentrations.

### 3.3. Comparing Noncompartmental Analysis and Population Pharmacokinetics Results

The results of the descriptive statistical analysis for the PK parameters of each subject, as calculated using NCA and population PK analyses, are listed in Table 3. Following the administration of 200 mg of teicoplanin intravenously, the mean CV% for AUC_inf_ and t_1/2λz_ were 307 (15.4%) mg/L·h and 54.6 (13.2%) h, respectively. The number of points used to calculate the t_1/2λz_, derived from the last measured concentration, consisted of either three or four measurements: four concentration measurements were taken for nine subjects and three for the remaining three subjects. The values for t_1/2λz_ significantly diverged from the three half-lives obtained through population PK analysis. The CL_NCA_ and AUC_inf_ calculated using NCA had values similar to the CL and AUC_tau_ derived from population PK, respectively; however, the steady-state volume of distribution from NCA (V_ssNCA_) significantly differed from that determined by population PK (V_SS_).

### 3.4. Dosage Simulations

**Figure 2:**
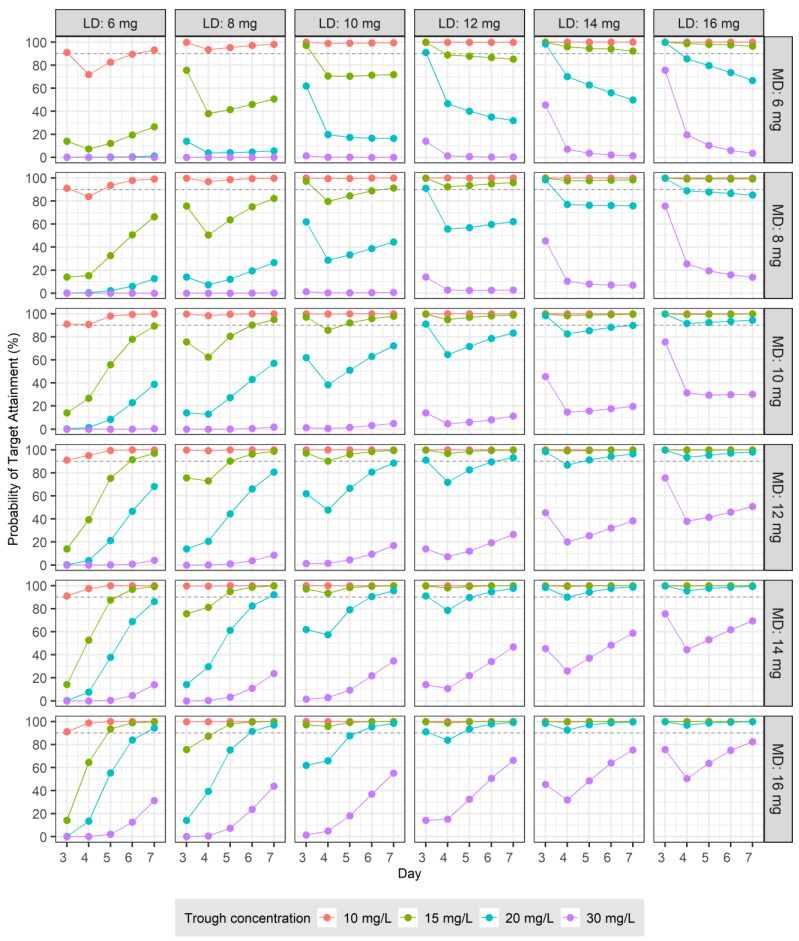
Probabilities of target trough concentrations (10, 15, 20, and 30 mg/L) in subjects with normal renal function: six loading doses (6, 8, 10, 12, 14, and 16 mg) were administered every 12 h four times, followed by daily maintenance doses (6, 8, 10, 12, 14, and 16 mg) from days 3 to 7.

**Figure 3:**
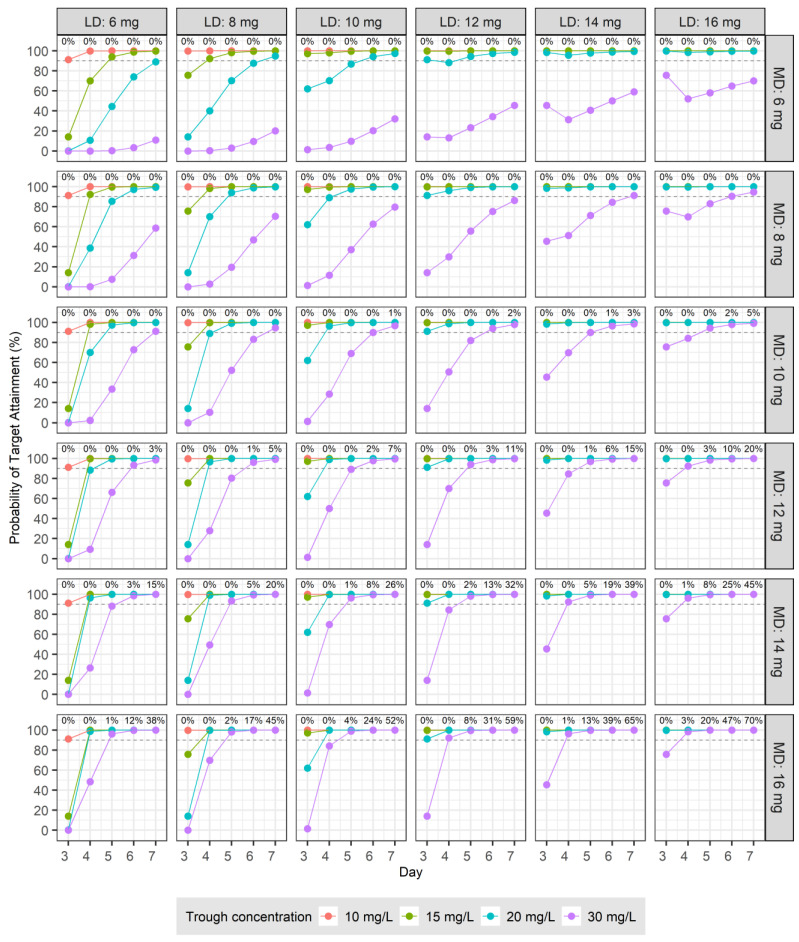
Probabilities of target trough concentrations (10, 15, 20, and 30 mg/L) in subjects with normal renal function: six loading doses (6, 8, 10, 12, 14, and 16 mg) were administered every 12 h four times, followed by six maintenance doses (6, 8, 10, 12, 14, and 16 mg) every 12 h ten times from day 3 to day 7. Each panel shows the proportion of cases in which the trough concentration exceeds 60 mg/L.

The therapeutic target was set to the C_trough_ of 10, 15, 20, or 30 mg/L for days 3–7 after the initiation of drug administration. The PTA for the loading and MDs administered at 12 and 24 h intervals are shown in Figure 2. The PTA derived from administrations at 12 h intervals for LDs and MDs are shown in Figure 3. For patients with normal renal function, an LD of 6 mg/kg at 12 h intervals for four doses followed by an MD of ≥10 mg/kg at 24 h intervals resulted in a PTA of ≥90% for a therapeutic target of C_trough_ > 10 mg/L from days 3 to 7 after the start of dosing (Figure 2). When the same patients were treated at the same intervals, but for a targeted C_trough_ > 20 mg/L, the LDs and MDs had to be over 14 mg/kg to achieve a PTA of ≥90% (Figure 2). For patients with normal renal function, an LD of 10 mg/kg at 12 h intervals for four doses followed by an MD of over 6 mg/kg at 12 h intervals resulted in a PTA of ≥90% for a C_trough_ > 15 mg/L for days 3–7 (Figure 3). For the same patients, treated at the same intervals, but aiming for a therapeutic target of C_trough_ > 30 mg/L, even with a 16 mg/kg LD, the PTA was <90% on day 3, and an MD ≥ 12 mg/kg was required to achieve a PTA of ≥90% from day 4 onward (Figure 3). For the majority of cases in which the MD was administered at 24 h intervals, the proportion of instances of C_trough_ > 60 mg/L was 0, and only a few exhibited values less than 1%. However, when administered at 12 h intervals, the proportion of instances of C_trough_ > 60 mg/L increased as the LD or MD increased. These values are displayed at the top of each panel in Figure 3.

**Figure 4:**
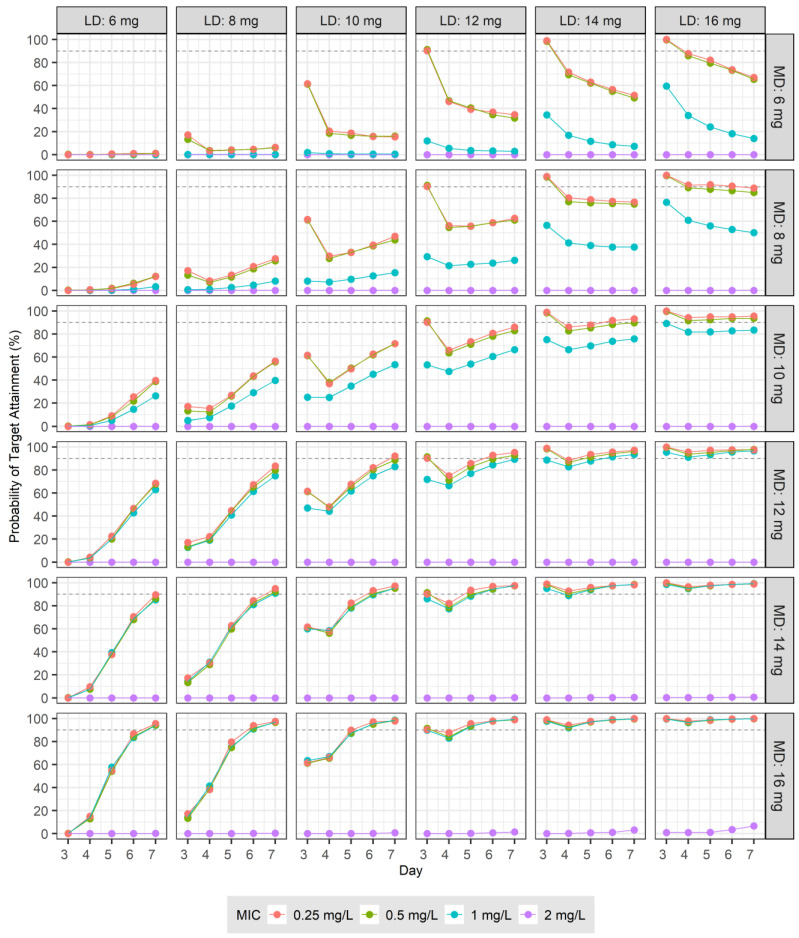
Probabilities of target attainment for trough concentrations >20 mg/L and AUC/MIC ≥ 800 in subjects with normal renal function across four MICs (0.25, 0.5, 1, and 2 mg/L): six loading doses (6, 8, 10, 12, 14, and 16 mg) were administered every 12 h four times, followed by six maintenance doses (6, 8, 10, 12, 14, and 16 mg) daily from days 3 to 7.

**Figure 5:**
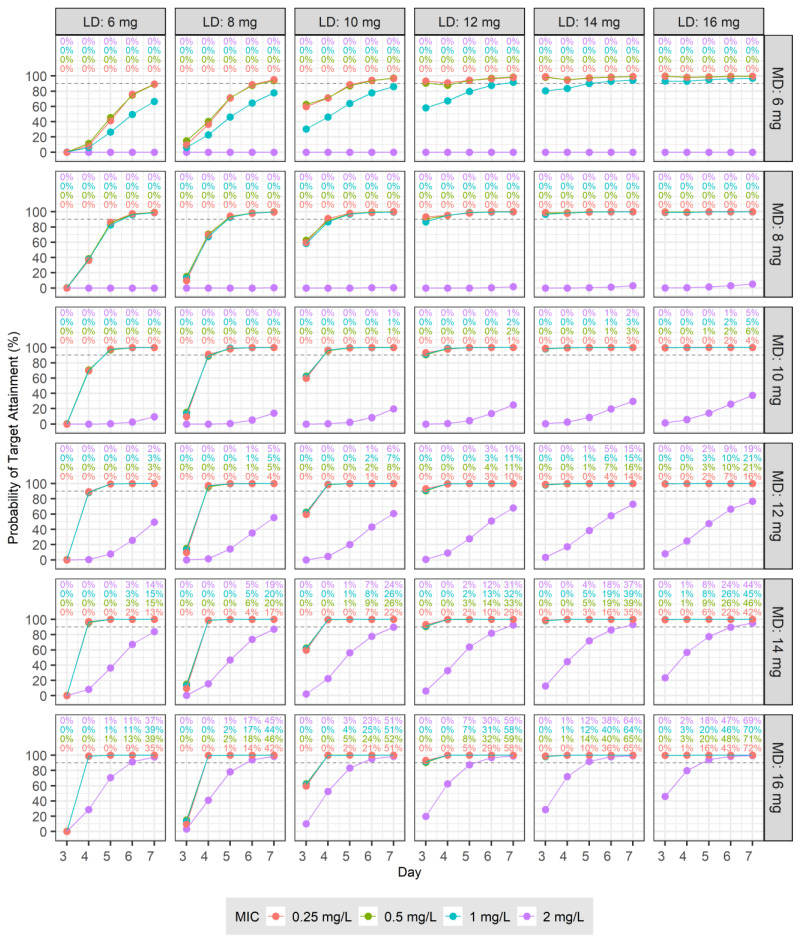
Probabilities of target attainment for trough concentrations >20 mg/L and AUC/MIC ≥800 in subjects with normal renal function across four MICs (0.25, 0.5, 1, and 2 mg/L): six loading doses (6, 8, 10, 12, 14, and 16 mg) administered every 12 h four times, followed by six maintenance doses (6, 8, 10, 12, 14, 16 mg) daily every 12 h 10 times from days 3 to 7. At the top of each panel, the proportion of cases in which the trough concentration exceeds 60 mg/L is shown.

The therapeutic targets were set to a C_trough_ > 20 mg/L and an AUC/MIC ≥ 800, and the PTA for various LDs and MDs administered at 12 and 24 h intervals are presented in Figure 4. The PTA for administrations at 12 h intervals for both LDs and MDs are shown in Figure 5. In patients with normal renal function who were infected with a pathogen with an MIC of 0.25 mg/L, administering an LD of 16 mg/kg at 12 h intervals for four doses followed by an MD of ≥10 mg/kg at 24 h intervals resulted in a PTA of ≥90% for the therapeutic target from days 3 to 7 after the start of dosing (Figure 4). For the same patients infected with a pathogen with an MIC of 1 mg/L, administering an LD of 16 mg/kg at 12 h intervals for four doses followed by an MD ≥ 12 mg/kg at 24 h intervals achieved a PTA of ≥90% for the therapeutic target from days 3 to 7 (Figure 4). In patients with normal renal function infected with a pathogen with an MIC of 0.5 mg/L, administering an LD of 12 mg/kg at 12 h intervals for four doses, followed by an MD of over 8 mg/kg at 12 h intervals, resulted in a PTA of ≥90% for the therapeutic target from days 3 to 7 (Figure 5). For the same patients infected with a pathogen with an MIC of 2 mg/L, even when an LD of 16 mg/kg and an MD of 16 mg/kg were administered at the same intervals, the PTA was <90% on days 3 and 4, with a PTA of ≥90% achieved starting from day 5 (Figure 5). When the MD was administered at 24 h intervals, the proportion of instances with C_trough_ > 60 mg/L was mostly 0 or near 0; however, at 12 h intervals, when either the LD or MD increased, the proportion of instances with C_trough_ > 60 mg/L also increased. These values are shown at the top of each panel in Figure 5 based on the MIC of the pathogen.

## 4. Discussion

Understanding the notion of normalcy is important for efficiently addressing abnormal conditions, as the objective of treatment is to revert the abnormal state to one that closely mirrors or aligns with the normal situation. In South Korea, there has yet to be a population PK study carried out for teicoplanin in healthy adults. To establish teicoplanin PK under the normal physiological conditions for healthy adults, we embarked on this study. Our goal was to develop a model with excellent predictive power by initially focusing on the PK of teicoplanin in healthy adults, and then applying this model to future patient-centered PK studies.

In the present study, the PK profile of teicoplanin in healthy adults was characterized using a three-compartment model. Typically, in studies with sparse sampling designs, the PK profile of teicoplanin is often delineated using one- [15,16] or two-compartment models [17,18]; however, previous studies using dense sampling approaches have also applied a three-compartment model [1,2,19,20]. Such models are characterized by three distinct slopes when plotted on a graph with the y-axis representing a logarithmic scale, corresponding to the composition of three exponential equations on a normal scale. Because of the potential risk for significant inaccuracies and imprecision in estimated PK parameters and predicted PK/PD indices with structural PK models that use fewer compartments based on sparse sampling [21,22], our version of the three-compartment model was effective at mitigating these potential risks. This model more accurately captures the PK profile, leading to more reliable and precise characterizations of teicoplanin behavior in the body. Unfortunately, studies establishing three-compartment models using a nonlinear mixed effect modeling approach are uncommon. For our population PK analysis, the typical values of CL and the steady-state volume of distribution (V_SS_, V1 + V2 + V3) for teicoplanin in healthy subjects were 0.693 L/h and 82.0 L, respectively (Table 2). In a study by Byrne et al. on 30 adult patients with hematological malignancy, CL and V_SS_ were 0.490 L/h and 81.3 L, respectively, with V1, V2, and V3 values of 4.32, 8.35, and 68.6 L, which showed a volume of distribution very similar to that in our study [19]. The patients in Byrne’s study were on average 64 years old, with a weight of 69.1 kg, and a CL_CR_ of 72 mL/min. Although the weight was similar to that in our cohort, the differences in the typical CL value appear to be primarily the results of differences in renal function and age.

We thoroughly examined various formulas for assessing renal function to determine which was the most suitable for explaining teicoplanin clearance. Through our analysis, we determined that eGFR, which was calculated using the CKD-EPI formula incorporating creatinine, but not cystatin C, and adjusted for each subject’s BSA (eGFR_CE1_), significantly affected teicoplanin clearance in our final model. To determine the rationale behind selecting this covariate, we compared estimated renal function as derived from different formulas to determine whether differences existed among them. The eGFR calculated using the MDRD and CKD-EPI formulas showed no significant differences before and after adjustment for body surface area (BSA). The eGFR values were calculated using the CKD-EPI formula with both creatinine and cystatin C, adjusted for BSA (eGFR_CE2_). Both the eGFR_CE1_ and eGFR_CE2_ demonstrated Shapiro–Wilk normality with *p*-values of 0.4574 and 0.8314, respectively. Similarly, the eGFR derived from the MDRD formula and adjusted for BSA (eGFR_M_) along with CL_CR_ also passed normality tests, with *p*-values of 0.3959 and 0.5871, respectively. Significant differences were observed between eGFR_CE1_ and eGFR_M_ in the *t*-test (*p* = 0.01995), and between eGFR_CE2_ and eGFR_M_ (*p* = 0.002735). There was no significant difference between CL_CR_ and eGFR_M_ (*p* = 0.05788). When CL_CR_, eGFR_CE1_, and eGFR_CE2_ were each compared using *t*-tests, no significant differences were observed. Interestingly, this is consistent with a previous study suggesting that the CKD-EPI formula may offer superior performance compared with the MDRD study equation, particularly at higher GFR levels [23].

Diverse criteria associated with favorable clinical responses have been proposed as therapeutic targets for teicoplanin, which vary based on the diagnosis. Using Monte Carlo simulation, we presented dosage regimens suitable for these diverse criteria when renal function is normal. In the first simulation, we calculated the PTA for various LD and MD regimens using C_trough_. These criteria were determined based on studies using C_trough_ as the therapeutic target, in which the PTA for various dosage regimens was evaluated for C_trough_ levels of 10, 15, 20, and 15–30 mg/L between days 2 and 4 after the start of treatment [24,25,26,27,28]. In studies examining the PK of teicoplanin and dose efficacy, diverse patient renal functions and C_trough_ targets have been examined. Mimoz et al. found that patients had a median (range) CL_CR_ of 113 (65–217) mL/min and a steady-state teicoplanin C_trough_ median (range) of 15.9 (8.8–29.9) mg/L. Their results supported a regimen of 12 mg/kg administered four times every 12 h, followed by a daily dose of 12 mg/kg as effective for treating ventilator-associated pneumonia caused by Gram-positive cocci [24]. Similarly, Ueda et al. found that in patients with a CL_CR_ > 90 mL/min, achieving a C_trough_ between 15 and 30 mg/L required an LD of 10–12 mg/kg twice daily for the initial 2 days, followed by 10–12 mg/kg once daily on the third day [26]. The results of Kato et al. further complement these dosing strategies, indicating that for patients with an eGFR > 80 mL/min/1.73 m^2^, a two-day high-dose regimen (40 mg/kg for 2 days) is sufficient to reach a C_trough_ of 15–30 mg/L by day 3 [28]. Similar to previous studies, in patients with normal renal function, administering 10 mg/kg every 12 h for four doses, followed by 12 mg/kg every 24 h, resulted in over 90% of patients achieving a target C_trough_ > 15 mg/L from day 3 onward (Figure 2). In the present study, to consistently achieve a target C_trough_ > 20 mg/L from day 3 onward in over 90% of patients, it was necessary to administer four LD of 12 mg/kg at 12 h intervals followed by an MD of at least 8 mg/kg at 12 h intervals. An important consideration is that when administering an MD of 12 mg/kg at 12 h intervals, up to 20% of patients reached a C_trough_ > 60 mg/L by day 7, which indicates the need for therapeutic drug monitoring (TDM) and dose adjustment during treatment (Figure 3).

In the second simulation, the PTA was determined for different LD and MD regimens to satisfy both criteria: a C_trough_ greater than 20 mg/L and an AUC/MIC ratio of at least 800. Adopting a similar approach to the first simulation, we used two distinct LD and MD combinations. The proportion of patients achieving both therapeutic targets was continuously assessed from days 3 to 7 following the initiation of treatment. In a study by Hagihara et al., ICU patients infected with MRSA with an MIC ≤ 1 mg/L and serum creatinine level < 1.5 mg/dL were administered 1200 mg on day 1, 1200 mg on day 2, and 600 mg on day 3. As a result, 100% of the patients achieved an AUC of ≥800 mg/L·h by day 3 [6]. Byrne’s study indicated that for the patients with a CL_CR_ of 120 mL/min, to achieve a C_trough_ > 20 mg/L at 72 h and on day 7 in over 90% of patients, an LD of 18 mg/kg every 12 h for five doses, followed by a daily MD of 12 mg/kg, is required. Meanwhile, for patients weighing 70 kg with a CL_CR_ of 70 mL/min and a pathogen MIC of 1 mg/L, administering an LD of 20 mg/kg every 12 h for five doses resulted in ≥90% of the patients achieving an AUC (from 48 to 72 h)/MIC ratio of ≥800 [19]. In the present study, which set two targets, a similar dosage regimen was required to achieve the goals in ≥90% of the patients. For pathogens with an MIC of 1 mg/L, administering an LD of 14 mg/kg every 12 h for four doses and an MD of 16 mg/kg every 24 h was required (Figure 4). For the same patients, when administering the MD every 12 h, a dose of 8 mg/kg was sufficient (Figure 5). For cases in which the MIC was 2 mg/L, increasing the dose frequency to every 12 h achieved a PTA of ≥90% between days 5 and 7; however, the proportion of patients with a C_trough_ > 60 mg/L gradually increased over time, suggesting that TDM and dose adjustment may be required for consistent treatment.

This study had several limitations. First, a small sample size of 12 subjects was insufficient to detect a variety of meaningful covariates, although eGFR and body weight were identified as significant factors. Thus, future studies with larger patient cohorts will be needed to develop more robust models. Second, we fixed the BSV for CL, V1, V2, and V3 due to their RSE values exceeding 25%, reflecting significant uncertainty. This decision, aimed at enhancing model stability and interpretability, involved employing the FOCE-I method and facing challenges with other estimation methods due to our dataset’s limitations and model complexity. It is important to note that excluding the BSV for these parameters significantly increased the OFV, thereby deteriorating the model’s fit to the data. Third, the administration of a single dose of teicoplanin raises concerns regarding the extrapolation and generalization to other doses and regimens; however, our simulations, ranging from 6 to 16 mg/kg, were strategically within teicoplanin’s linear PK range of 2–25 mg/kg [3]. Fourth, this study establishes a foundational population PK model for healthy adults but acknowledges the limitation of not covering all adult age groups and ethnicities. This could limit the model’s broader applicability, particularly among geriatric and frail elderly populations with distinct PK profiles due to aging and comorbidities. Also, our focus on healthy individuals means we miss direct microbiological or clinical outcome assessments, essential for model validation in therapeutic dose prediction. Moving forward, we aim to refine our model by integrating broader demographic data from patient-based studies, enhancing its predictive accuracy and clinical relevance. Our goal is to develop a model adaptable to diverse patient populations, thus broadening its utility in designing optimal dosage regimens and improving therapeutic outcomes.

In conclusion, we established PK properties for teicoplanin in healthy subjects by applying an NCA and a population approach. The concentration–time profile of teicoplanin is explained using a three-compartment model. Results from Monte Carlo simulations suggest that, in patients with normal renal function, an increase in both LDs and MDs, or a decrease in the interval of MDs, should be considered. Specifically, for pathogens with an MIC of 1 mg/L, we recommend administering an LD of 14 mg/kg every 12 h for four doses, followed by an MD of 16 mg/kg every 24 h. However, because of the long half-life of teicoplanin, in cases requiring long-term administration, it is necessary to perform TDM at appropriate times to prevent nephrotoxicity resulting from a high C_trough_.

## Figures and Tables

**Table 1:** Subject characteristics.

Parameters	Mean (CV%.)	Median (IQR)
Demographic characteristics		
Age, years	34.8 (17.2%)	32.0 (30.0–40.3)
Height, cm	165 (4.91%)	164 (158–169)
Weight, kg	64.8 (19.9%)	67.9 (51.3–73.4)
Body surface area, m^2^	1.71 (11.3%)	1.77 (1.52–1.85)
Body mass index (BSA), kg/m^2^	23.6 (15.1%)	24.8 (20.6–25.5)
Laboratory characteristics		
Protein, g/dL	7.52 (5.07%)	7.35 (7.30–7.70)
Albumin, g/dL	4.96 (4.90%)	4.90 (4.80–5.03)
Cystatin C, mg/dL	0.808 (14.6%)	0.760 (0.710–0.928)
Creatinine, mg/dL	0.867 (15.2%)	0.875 (0.793–0.925)
Blood urea nitrogen, mg/dL	11.2 (26.0%)	11.2 (10.1–12.0)
Alanine aminotransferase, U/L	23.2 (79.7%)	19.5 (8.50–29.0)
Aspartate aminotransferase, U/L	25.9 (41.3%)	22.5 (19.5–27.5)
Gamma-glutamyl transferase, U/L	23.8 (46.6%)	22.5 (17.0–28.0)
Renal functions		
CLCR by Cockcroft–Gault (mL/min) ^a^	102 (19.5%)	104 (94.3–113)
eGFR by MDRD (mL/min/1.73 m^2^) ^b^	88.6 (13.4%)	89.4 (81.8–96.1)
eGFR by CKD-EPICR (mL/min/1.73 m^2^) ^c^	104 (10.7%)	105 (97.7–115)
eGFR by CKD-EPICR-CYS (mL/min/1.73 m^2^) ^d^	108 (11.6%)	108 (98.8–120)
Adjusted eGFR by MDRD for BSA (mL/min) ^e^	87.5 (16.3%)	89.7 (80.1–96.0)
Adjusted eGFR by CKD-EPICR for BSA (mL/min) ^e^	103 (15.5%)	105 (96.7–113)
Adjusted eGFR by CKD-EPICR-CYS for BSA (mL/min) ^e^	106 (11.8%)	105 (101–112)

CV, coefficient of variation; IQR, interquartile range; CL_CR_, creatinine clearance; eGFR, estimated glomerular filtration rate; MDRD, modification of diet in renal disease; CKD-EPI, chronic kidney disease epidemiology collaboration; CR, creatinine; CYS, cystatin C; min, the minimum of (CR or CYS)/number and 1; max, the maximum of (CR or CYS)/number and 1. ^a^ CL_CR_ = (140 − Age) × weight/CR × 72 (×0.85 if female). ^b^ eGFR = 175 × CR^−1.154^ × Age^−0.203^ × (0.742 if female). ^c^ eGFR (female) = 142 × min(CR/0.7,1)^−0.241^ × max(CR/0.7,1)^−1.200^ × 0.9938^Age^ × ^1.0^12, e GFR (male) = 142 × min(CR/0.9,1)^−0.302^ × max(CR/0.9,1)^−1.200^ × 0.9938^Age^. ^d^ eGFR (female) = 135 × min(CR/0.7,1)^−0.219^ × max(CR/0.7,1)^−0.544^ × min(CYS/0.8,1)^0.323^ × max(CYS/0.8,1)^−0.778^ × 0.9961^Age^ × 0.963, eGFR (male) = 135 × min(CR/0.9,1)^−0.144^ × max(CR/0.9,1)^−0.544^ × min(CYS/0.8,1)^0.323^ × max(CYS/0.8,1)^−0.778^ × 0.9961^Age^. ^e^ The adjusted eGFR by MDRD and CKD-EPI equations are eGFR = eGFR (MDRD or CKD-EPI) × (BSA/1.73 m^2^).

**Table 2:** Parameter estimates and bootstrap medians (95% confidence intervals) for the final pharmacokinetic model of teicoplanin in 12 healthy adult subjects.

Parameter	Estimates	RSE(%)	Bootstrap Median (95% CI)
Structural model			
CL = θ_1_ × (CE/105.27) ^θ2^			
θ_1_ (L/h)	0.693	2.97	0.693 (0.653–0.74)
θ_2_	0.785	16.2	0.789 (0.422–1.16)
V1 = θ_3_ (L)	3.96	8.41	3.97 (3.15–4.62)
Q2 = θ_4_ (L/h)	4.45	11.6	4.45 (3.63–5.86)
V2 = θ_5_ (L)	8.24	8.32	8.33 (7.07–9.85)
Q3 = θ_6_ (L/h)	1.76	9.7	1.75 (1.44–2.13)
V3 = θ_7_ × (WT/67.85) ^θ8^			
θ_7_ (L)	69.8	8.74	69.7 (55.8–82.6)
θ_8_	1.73	22.6	1.73 (0.67–2.44)
Interindividual variability			
CL (%)	8.83 ^f^		
V1 (%)	23.8 ^f^		
Q2 (%)	32.7	20.2	30.7 (14.2–42.2)
V2 (%)	23.9 ^f^		
Q3 (%)	31.0	18.9	29.6 (16.7–41.4)
V3 (%)	7.54 ^f^		
Residual variability			
Proportional error (%)	6.33	13.1	6.22 (4.63–7.94)

RSE, relative standard error; CI, confidence interval; CL, total clearance; V1, central volume of distribution; V2, the volume of distribution for the first peripheral compartment; Q2, intercompartmental clearance between V1 and V2; V3, the volume of distribution for the second peripheral compartment; Q3, intercompartmental clearance between V1 and V3; CE, estimated glomerular filtration rate calculated using the CKD-EPI equation based on creatinine levels, adjusted for body surface area; WT, weight; ^f^, fixed.

**Table 3:** Descriptive statistics of individual pharmacokinetic parameters from noncompartmental and population PK analysis.

Parameters	Unit	Mean (CV%)	Median (IQR)
NCA results			
C_max_	mg/L	32.1 (12.9%)	30.9 (29.5–35.6)
T_last_	h	187 (11.0%)	192 (169–194)
C_last_	mg/L	0.431 (24.9%)	0.422 (0.348–0.470)
AUC_last_	mg/L·h	273 (16.6%)	270 (247–302)
AUC_inf_	mg/L·h	307 (15.4%)	305 (279–332)
AUMC_last_	mg/L·h^2^	13,800 (16.0%)	13,800 (12,300–15,000)
AUMC_inf_	mg/L·h^2^	22,700 (14.8%)	22,300 (20,200–23,200)
MRT_inf_	h	74.0 (12.8%)	76.7 (68.0–80.7)
CL_NCA_	L/h/kg	0.0105 (15.2%)	0.0103 (0.00896–0.0117)
V_zZNCA_	L/kg	0.825 (21.3%)	0.761 (0.693–0.891)
V_ssNCA_	L/kg	0.776 (19.6%)	0.734 (0.661–0.859)
t_1/2λz_	h	54.6 (13.2%)	55.0 (51.9–58.6)
Population PK results			
CL	L/h/kg	0.0107 (14.0%)	0.0104 (0.00961–0.0122)
V_C_	L/kg	0.0644 (28.9%)	0.0649 (0.0526–0.0733)
V_ss_	L/kg	1.21 (23.0%)	1.24 (1.06–1.34)
AUC	mg/L·h	299 (16.0%)	300 (263–316)
1st t_1/2_	h	0.345 (36.3%)	0.317 (0.289–0.417)
2nd t_1/2_	h	4.12 (22.2%)	4.23 (3.78–4.83)
3rd t_1/2_	h	103 (29.9%)	97.9 (83.1–116)

CV, coefficient of variation; IQR, interquartile range; C_max_, maximum observed plasma concentration; T_last_, time of last measurable concentration; C_last_, concentration corresponding to T_last_; AUC_last_, area under the plasma concentration–time curve (AUC) from the start of dosing to the last quantifiable concentration; AUC_inf_, AUC from the start of dosing to infinity; AUMC_last_, area under the first moment curve (AUMC) from 0 h to the T_last_; AUMC_inf_, AUMC extrapolated to infinity, based on the last observed concentration; MRT_inf_, mean residence time from 0 h to infinite; CL_NCA_, total body clearance determined using NCA; V_zNCA_, volume of distribution (Vd) determined using NCA; V_ssNCA_, steady-state Vd determined using NCA; t_1/2λz_, terminal elimination half-life; AUC_inf_, AUC_last_ + C_last_/λ_z_; AUMC_inf_, AUMClast + (T_last_ × Clast)/λ_z_ + Clast/λ_z_^2^; MRT_inf_, AUMC_inf_/AUC_inf_—infusion time/2; CL_NCA_, dose/AUC_inf_; V_zNCA_, CL_NCA_/λ_z_; V_ssNCA_, MRT_inf_ × CL_NCA_; t_1/2λz_, ln(2)/λ_z_; CL, total clearance; V_C_, central volume of distribution; V_ss_, steady-state volume of distribution; AUC, dose/CL; 1st–3rd t_1/2_, three half-lives for the final three-compartment model.

## Data Availability

The datasets generated and/or analyzed during the current study are available from the corresponding author upon reasonable request.

## Supplementary Materials

The following supporting information can be downloaded at: https://www.mdpi.com/article/10.3390/pharmaceutics16040499/s1, Figure S1. Individual fit plots of Teicoplanin (a) normal scale, (b) semi-log scale: closed circles, observed concentrations; solid line, individual-predicted concentrations; dotted line, population-predicted concentrations. Figure S2. Visual predictive check from simulated concentrations of 1000 virtual datasets of teicoplanin (a) 0 to 4 h, (b) 0 to 240 h: closed circles, observed concentrations; solid lines, 10th, 50th and 90th percentiles of observations; dashed lines, 10th, 50th and 90th percentiles of simulated concentrations; and shaded areas, 95% confidence intervals for the 10th, 50th, and 90th percentiles of simulated concentrations.
