# Supplementary material for: Beyond One-Size-Fits-All: Tailoring Teicoplanin Regimens for Normal Renal Function Patients Using Population Pharmacokinetics and Monte Carlo Simulation
Source: Pharmaceutics. 2024 Apr 5;16(4):499. doi: 10.3390/pharmaceutics16040499 (PMC11053983; doi:10.3390/pharmaceutics16040499)
Supplement: Supplementary file 1 [file pharmaceutics-16-00499-s001.zip › pharmaceutics-2946485-supplementary.pdf]

Supplementary Figures

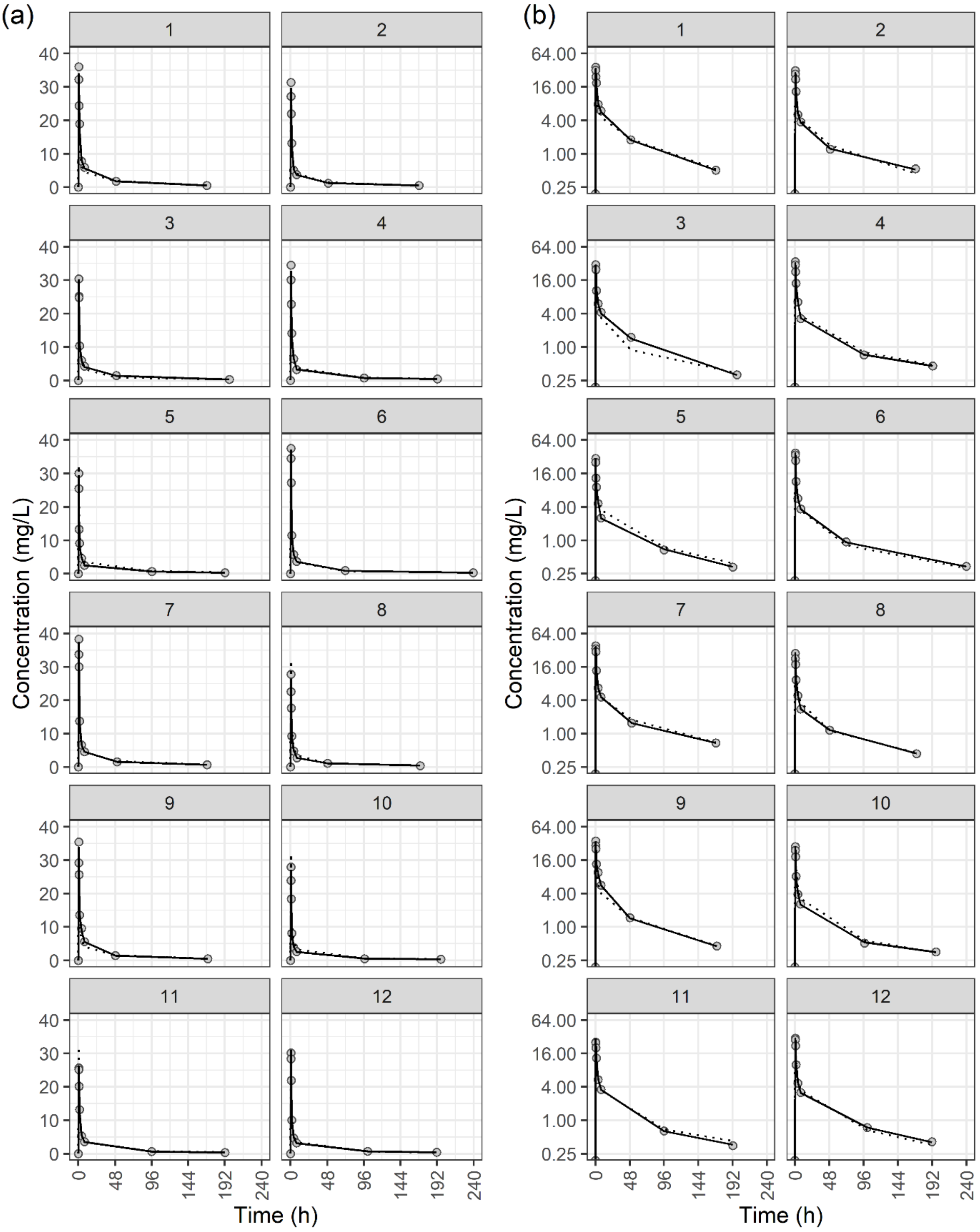

Figure S1. Individual fit plots of Teicoplanin (a) Normal scale, (b) semi-log scale: closed circles, observed concentrations; solid line, individual-predicted concentrations; dotted line, population-predicted concentrations.

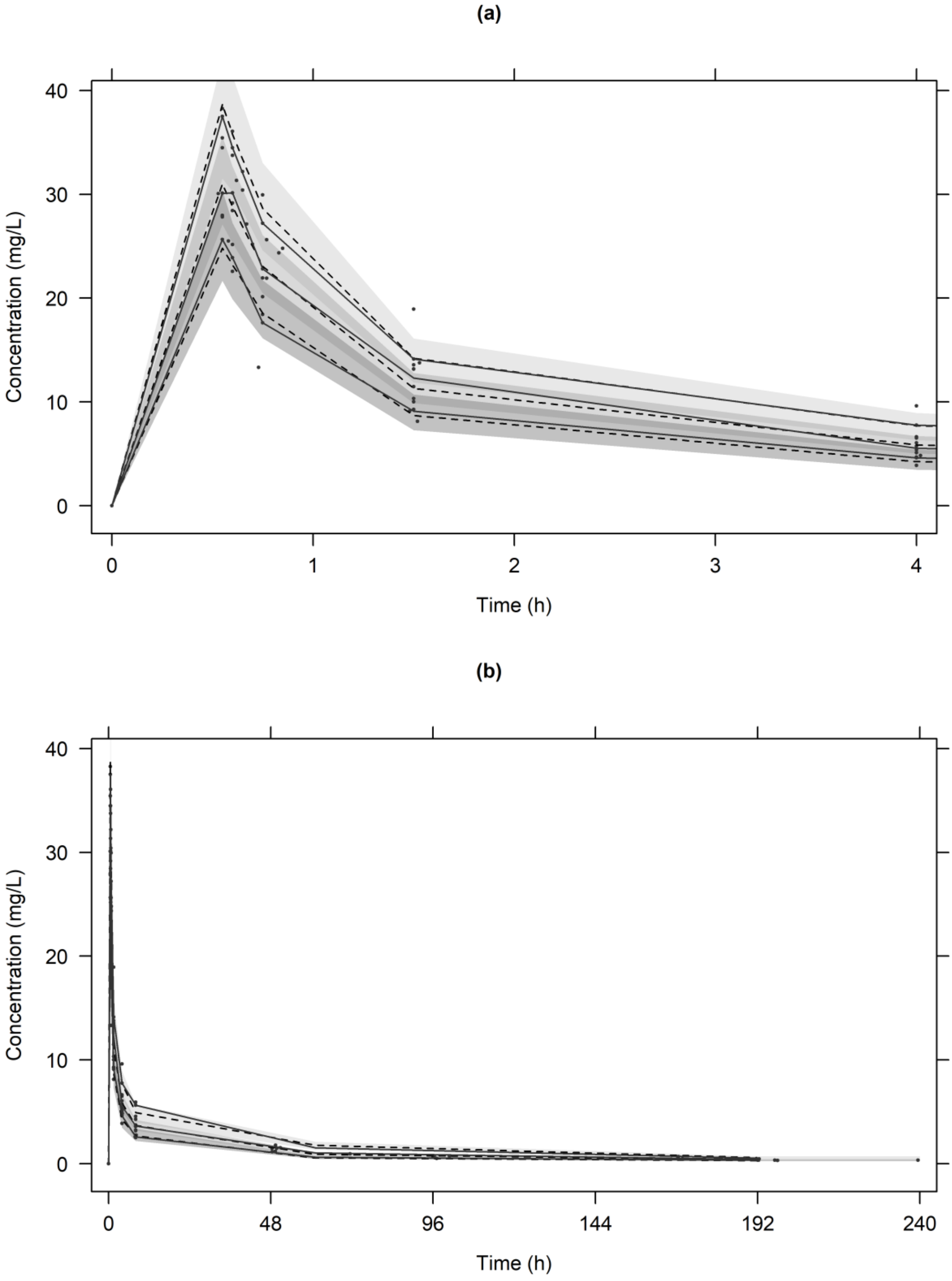

Figure S2. Visual predictive check from simulated concentrations of 1,000 virtual datasets of teicoplanin (a) 0 to 4 h, (b) 0 to 240 h: closed circles, observed concentrations; solid lines, 10th, 50th and 90th percentiles of observations; dashed lines, 10th, 50th and 90th percentiles of simulated concentrations; and shaded areas, 95% confidence intervals for the 10th, 50th, and 90th percentiles of simulated concentrations.
